# Reply to the comment

**DOI:** 10.1186/s40560-020-00438-3

**Published:** 2020-03-13

**Authors:** Yohei Okada, Takeshi Unoki, Yujiro Matsuishi, Yuko Egawa, Kei Hayashida, Shigeaki Inoue

**Affiliations:** 1 Department of Primary Care and Emergency Medicine, Graduate School of Medicine, Kyoto University, Syogoin Kawaramachi 54, Sakyo, Kyoto, 606-8507 Japan; 2 Preventive Services, School of Public Health in the Graduate School of Medicine, Kyoto University, Kyoto, Japan; 3 School of Nursing, Sapporo City University, Sapporo, Japan; 4 Emergency and Intensive Care Laboratory, University of Tsukuba, Ibaraki, Japan; 5 Pediatric Intensive Care Unit, University of Tsukuba Hospital, Ibaraki, Japan; 6 Advanced Emergency and Critical Care Center, Saitama Red Cross Hospital, Saitama, Japan; 7 The Feinstein Institutes for Medical Research, Department of Emergency Med-Cardiopulmonary, North Shore University Hospital, Northwell Health System, New York, USA; 8 Department of Disaster and Emergency Medicine, Graduate School of Medicine, Kobe University, Kobe, Japan

We would like to thank you for the opportunity to respond to the comment from Dr. K Friedrich Kuhn on our manuscript [1]. Initially, we clarify the outline of our systematic review. We included randomized controlled trials which compared early mobilization defined as started within 1 week of intensive care unit admission and earlier than usual care or control, with the control defined as usual care or mobilization initiated later than the intervention (not “later than 1 week” as described in the comment). The description in the comment was incorrect. As the primary outcomes, we focused on the mortality and health-related quality of life (HRQOL).

Basically, we agree that the timing of early mobilization is one of the most significant components to be discussed, and it is a problem that there is no uniform definition of “early mobilization.” Although we did not perform subgroup analysis to identify the heterogeneity based on the timing, it may be reasonable that the different effect would exist between the groups with different timing.

However, as our study indicated [2], we believe that there is no apparent evidence to support that early mobilization can improve mortality or HRQOL. In the comments, they introduced some studies with different timing of early mobilization, and these studies indicated the effects on some outcomes; however, these studies did not show the impact neither on mortality nor on HRQOL. Only one [3] suggests the improvement of the HRQOL; but it was assessed as high risk of bias in some domains in our systematic review [2]. Therefore, we believe that it is unclear whether the early mobilization can improve mortality or HRQOL regardless of the difference of the definition based on the current available evidence.

## Data Availability

Not applicable
